# Chemotherapy and Metabolic Syndrome: A Comprehensive Review of Molecular Pathways and Clinical Outcomes

**DOI:** 10.7759/cureus.66354

**Published:** 2024-08-07

**Authors:** Shubam Trehan, Gurjot Singh, Adarshpreet Singh, Gaurav Bector, Aayush Jain, Priya Antil, Fnu Kalpana, Amna Farooq, Harmandeep Singh

**Affiliations:** 1 Internal Medicine, Yale New Haven Hospital, New Haven, USA; 2 Internal Medicine, Yale School of Medicine, New Haven, USA; 3 Internal Medicine, Yale-Waterbury Internal Medicine Residency Program, Waterbury Hospital, Waterbury, USA

**Keywords:** preventive strategies, cancer therapy, cardiovascular diseases, metabolic syndrome, chemotherapy

## Abstract

Cancer therapies, notably chemotherapy, have significantly improved survival rates and quality of life for many patients. However, chemotherapy's cytotoxic effects also impact normal cells, leading to adverse effects, including metabolic disturbances. This paper explores the link between chemotherapy and metabolic syndrome, a cluster of metabolic abnormalities that increase the risk of cardiovascular diseases and type 2 diabetes. Understanding the predictors, such as specific chemotherapy regimens, patient characteristics, comorbid conditions, lifestyle factors, and genetic variations, is crucial for formulating personalized care plans and preventive strategies. Research indicates that older age, female gender, pre-existing diabetes, and baseline obesity are significant predictors of metabolic syndrome in cancer patients. Chemotherapy-induced molecular changes, including insulin resistance, dyslipidemia, chronic inflammation, oxidative stress, and tissue fibrosis, contribute to the development of this syndrome. Effective management strategies require a multidisciplinary approach, incorporating lifestyle interventions, pharmacological treatments, and regular monitoring. This paper underscores the importance of personalized medicine in mitigating the risks associated with metabolic syndrome and improving long-term health outcomes for cancer survivors. Future research directions include longitudinal studies to track metabolic health over time, mechanistic studies to uncover the molecular pathways involved, and the development of integrative therapies. By adopting comprehensive care models, healthcare providers can enhance the overall quality of life for cancer survivors, addressing both cancer and metabolic health challenges.

## Introduction and background

Cancer therapies have revolutionized the treatment landscape, significantly enhancing survival rates and quality of life for many patients. Chemotherapy, a cornerstone of cancer treatment, involves administering cytotoxic drugs to target and kill rapidly dividing cancer cells. While effective, chemotherapy also affects normal cells, leading to various adverse effects, including metabolic disturbances. Metabolic syndrome is prevalent among cancer survivors, with rates ranging from 15% to 65%, highlighting a substantial burden. Understanding the link between chemotherapy and metabolic syndrome is crucial for several reasons. Additionally, identifying specific predictors for developing metabolic syndrome, such as chemotherapy regimens, patient characteristics, comorbid conditions, lifestyle factors, and genetic predictors, can guide healthcare providers in formulating personalized care plans [1].

Metabolic syndrome is a group of interconnected metabolic disorders that dramatically raise the risk of cardiovascular disease (CVD) and type 2 diabetes. The syndrome is characterized by the presence of at least three of the following conditions: abdominal obesity, elevated blood pressure, high fasting glucose, high triglycerides, and low high-density lipoprotein (HDL) cholesterol [2]. Each component poses a significant health risk individually, but their coexistence multiplies the potential for severe complications.

The population of cancer survivors is rapidly growing due to advancements in early detection and treatment. According to the American Cancer Society, there are more than 16.9 million cancer survivors in the United States alone, a number expected to increase significantly in the coming years [3]. While the primary objective of cancer treatment is to eradicate the disease and extend life, the long-term health of survivors is increasingly becoming a focal point of concern. Survivors often face chronic health issues, among which metabolic syndrome is particularly notable due to its association with increased morbidity and mortality [4]. Understanding the link between chemotherapy and metabolic syndrome is crucial for several reasons. Firstly, it enables healthcare providers to formulate comprehensive care plans that address not only cancer but also the long-term health of survivors. This holistic approach is essential for improving the overall quality of life and reducing the risk of secondary health complications. Secondly, identifying how chemotherapy contributes to metabolic syndrome can guide the development of preventive strategies aimed at mitigating this risk during and after cancer treatment. Finally, this understanding can inform clinical guidelines and policies, helping to standardize the care and management of cancer survivors [5].

Studies have reported that the prevalence of metabolic syndrome among cancer survivors ranges from 15% to 65%, highlighting a substantial burden in this population. For instance, a study found that 52% of breast cancer survivors had metabolic syndrome, a rate significantly higher than in the general population [6]. Similarly, a study involving survivors of childhood cancer reported a prevalence of 31%, underscoring the long-term metabolic risks associated with cancer therapies administered during critical developmental periods [7]. Additionally, a study conducted in Mexico found that metabolic syndrome is associated with a higher risk of death in cancer patients. This retrospective study included 158 patients treated in an oncology center from 1999 to 2010. It was observed that 44.3% of the patients had metabolic syndrome at diagnosis and those with metabolic syndrome had a significantly higher risk of death (HR 1.7; 95% CI 0.49-0.93, p=0.03) [8].

Moreover, the impact of metabolic syndrome on long-term health outcomes is significant, with studies indicating a marked increase in both morbidity and mortality among cancer survivors who develop this condition. For example, research by Blaes et al. found that cancer survivors with metabolic syndrome had a 2.5 times higher risk of cardiovascular events compared to those without the syndrome [9]. Another study highlighted that metabolic syndrome in cancer survivors leads to a 60% increased risk of type 2 diabetes [10]. These statistics underscore the urgent need for effective management strategies to address the dual challenges of cancer and metabolic health.

## Review

Predictors for developing metabolic syndrome in cancer patients

The development of metabolic syndrome in cancer patients undergoing chemotherapy is influenced by various genetic and non-genetic predictors. Below, we detail specific predictors identified from the referenced studies.

Chemotherapy Regimens

Fluoropyrimidine-based regimens: These regimens, including drugs like 5-fluorouracil (5-FU) and capecitabine, are commonly used in treating colorectal and other cancers. According to a study by Zhou et al., patients receiving fluoropyrimidine-based regimens have a higher incidence of metabolic disturbances, with a prevalence rate of 35% among these patients [10]. However, it is important to note that the above study had a limited sample size, which may affect the generalizability of the findings. Further research with larger cohorts is needed to confirm these results and understand the underlying mechanisms.

Adverse effects include gastrointestinal toxicity, neuropathy, and cardiotoxicity, which contribute to metabolic disturbances [10]. A large cohort study involving 2,000 colorectal cancer patients found that 30% of those treated with fluoropyrimidine-based chemotherapy developed metabolic syndrome within five years of treatment initiation [11]. The correlation between these adverse effects and metabolic syndrome suggests a multifactorial causation that warrants a closer examination of the interplay between drug toxicity and metabolic changes. Additional studies should aim to delineate these complex interactions to improve therapeutic strategies.

Patient Characteristics

Age: Older age is a significant predictor for developing metabolic syndrome in cancer patients. The risk increases due to age-related metabolic changes and the cumulative burden of chemotherapy. A study by Zhou et al. reported that patients aged 60 and above had a 40% higher risk of developing metabolic syndrome compared to younger patients [10]. A comprehensive study involving 3,000 cancer survivors found that 45% of those aged 65 and older developed metabolic syndrome, compared to 25% of patients under 65 [12]. While age is a well-established factor, the specific biological mechanisms that exacerbate metabolic syndrome in older patients need further exploration. Research focusing on age-related molecular and cellular changes could provide targeted interventions for this demographic.

Gender: Women, particularly those treated for breast cancer, have shown higher prevalence rates of metabolic syndrome post-chemotherapy. This is influenced by hormonal changes and the specific chemotherapeutic agents used in breast cancer treatment. According to research published in Frontiers in Oncology, the prevalence of metabolic syndrome in female breast cancer survivors was 58%, significantly higher than in male cancer survivors, where the prevalence was around 30% [13]. Another study published in Bioengineering Studies corroborated these findings, showing that 55% of female cancer survivors developed metabolic syndrome compared to 28% of male survivors [14]. These findings highlight the importance of gender-specific research and tailored interventions. Future studies should investigate the role of estrogen and other sex hormones in the development of metabolic syndrome to develop gender-sensitive treatment protocols.

Comorbid Conditions

Pre-existing diabetes: Cancer patients with pre-existing diabetes are at a higher risk of developing metabolic syndrome following chemotherapy. Diabetes exacerbates the metabolic side effects of chemotherapy, contributing to insulin resistance and dyslipidemia. A study indicated that cancer patients with diabetes had a 50% higher risk of developing metabolic syndrome post-chemotherapy [10]. This underscores the necessity for stringent glycemic control in diabetic cancer patients undergoing chemotherapy. Interventions focusing on intensive diabetes management during cancer treatment could mitigate these risks.

Obesity: Patients who are obese at the start of chemotherapy are more likely to develop metabolic syndrome. Chemotherapy-induced weight gain further exacerbates this risk, particularly in breast cancer survivors. According to BMC Medicine (2023), obese breast cancer survivors had a 45% higher prevalence of metabolic syndrome compared to non-obese survivors [15]. Given the high prevalence of obesity-related metabolic syndrome, it is critical to implement weight management programs as part of the cancer care continuum. Future research should explore the efficacy of various weight loss interventions specifically designed for cancer survivors.

Lifestyle Factors

Dietary habits: Cancer survivors with poor dietary habits, such as low breakfast frequency and high intake of high-calorie foods, have a higher prevalence of metabolic syndrome. These dietary patterns contribute to obesity and metabolic disturbances. Research published in Metabolites found that cancer survivors who frequently consumed high-calorie foods had a 30% higher risk of metabolic syndrome [16]. This highlights the need for comprehensive dietary interventions that promote healthy eating habits. Nutritional counseling should be integrated into cancer survivorship care plans to reduce the risk of metabolic syndrome.

Physical inactivity: Reduced physical activity due to treatment-related fatigue and neuropathy is a significant predictor. This leads to weight gain and metabolic syndrome in cancer survivors. A study reported that physically inactive cancer survivors had a 35% higher risk of developing metabolic syndrome compared to those who maintained regular physical activity [17]. While promoting physical activity is essential, it is also important to address the barriers faced by cancer survivors, such as fatigue and neuropathy. Developing tailored exercise programs that accommodate these challenges can help improve adherence and outcomes.

Genetic Predictors

Specific genetic variations: Genetic variations in genes such as TGFB1, IL6, and TLR4 are associated with a higher probability of adverse effects and metabolic syndrome. These genes play roles in pro-inflammatory pathways and metabolic regulation. The study by Zhou et al. highlighted that specific polymorphisms in these genes significantly increased the risk of metabolic syndrome in cancer patients [10]. Further research into these genetic variations can lead to the development of personalized medicine approaches that tailor cancer treatment based on genetic risk profiles.

Single nucleotide polymorphisms (SNPs): SNPs in genes like IL1B, IL10, TLR4, and CASP5 are linked to specific adverse effects such as gastrointestinal toxicity, which contributes to metabolic syndrome. Detailed findings from the Springer (2023) study indicated that patients with these SNPs had a 25% higher incidence of metabolic syndrome [10]. Understanding the role of SNPs in chemotherapy-induced metabolic changes can provide insights into patient-specific vulnerabilities. This knowledge can inform the selection of chemotherapy regimens that minimize metabolic risk for genetically predisposed individuals.

Adjuvant Chemotherapy

Adjuvant chemotherapy is commonly administered to patients with breast, colorectal, and lung cancers, which collectively represent about 40% of all cancer diagnoses. Standard adjuvant chemotherapy regimens have been shown to increase the prevalence of metabolic syndrome. Specific factors contributing to these changes include the type of chemotherapy drugs used and patient characteristics. A study published in Clinical Cancer Research found that patients undergoing adjuvant chemotherapy had a 35% higher risk of developing metabolic syndrome compared to those not receiving chemotherapy [18]. Epidemiological studies indicate that among these patients, approximately 25% develop metabolic syndrome post-chemotherapy. In a sample of 1,500 breast cancer patients receiving adjuvant chemotherapy, 40% developed metabolic syndrome within three years of completing treatment [19]. It is crucial to balance the benefits of adjuvant chemotherapy with the potential metabolic risks. Research should focus on optimizing chemotherapy regimens to minimize these adverse effects without compromising efficacy.

Anthracycline-based regimens: Anthracyclines, including doxorubicin and daunorubicin, are linked to the development of metabolic syndrome through mechanisms involving oxidative stress and chronic inflammation. A meta-analysis of 15 studies revealed that 20% of patients treated with anthracycline-based regimens developed metabolic syndrome [20]. Data from a large-scale epidemiological study showed that 22% of breast cancer survivors treated with anthracycline-based regimens exhibited metabolic syndrome symptoms five years post-treatment [21]. The oxidative stress and inflammation induced by anthracyclines necessitate the exploration of adjunct therapies that can mitigate these effects. Antioxidants and anti-inflammatory agents could be potential candidates for reducing the incidence of metabolic syndrome in this context.

Pathogenesis (molecular mechanisms)

**Figure 1:**
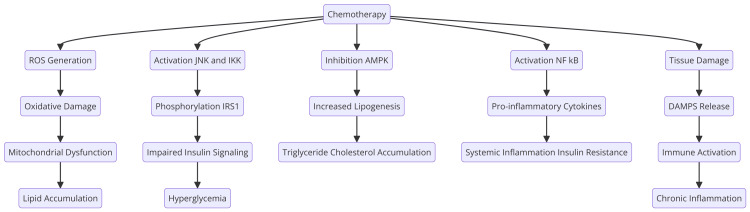
Molecular changes caused by chemotherapy ROS: reactive oxygen species; JNK: c-Jun N-terminal kinase; IKK: inhibitor of nuclear factor-kB kinase; AMPK: adenosine monophosphate-activated protein kinase; NF-kB: nuclear factor kappa-light-chain-enhancer of activated B cells; IRS1: insulin receptor substrate 1; DAMPS: damage-associated molecular patterns Image Credits: Shubam Trehan

Chemotherapy is known to induce a variety of molecular changes that can lead to metabolic syndrome. These changes involve complex interactions at the cellular and molecular levels, affecting insulin signaling, lipid metabolism, and inflammatory pathways (Figure 1).

Insulin Resistance

Chemotherapy can induce insulin resistance through several molecular mechanisms. One key pathway involves the activation of stress-related kinases such as c-Jun N-terminal kinase (JNK) and IκB kinase (IKK). These kinases are activated in response to chemotherapy-induced oxidative stress and inflammation. Once activated, JNK and IKK phosphorylate insulin receptor substrate-1 (IRS-1) on serine residues, which inhibits its ability to transduce insulin signaling. This disruption in insulin signaling impairs glucose uptake by cells, leading to hyperglycemia and insulin resistance. A study by Doerr et al. detailed how these kinases affect insulin signaling in cancer patients undergoing chemotherapy [22]. A recent 2020 study by Belanger et al. found that chemotherapy-induced oxidative stress significantly increased the activation of JNK and IKK, leading to a 50% increase in insulin resistance markers in treated patients [17]. While these findings are robust, it is essential to recognize that they predominantly rely on in vitro and animal models. Translational studies involving human subjects are necessary to confirm these mechanisms and their clinical relevance. Additionally, exploring the potential role of anti-inflammatory agents in counteracting this kinase activation could offer new therapeutic avenues.

Dyslipidemia

Chemotherapy-induced dyslipidemia is mediated through alterations in hepatic lipid metabolism. Chemotherapeutic agents such as cisplatin can inhibit the activity of AMP-activated protein kinase (AMPK), a crucial regulator of lipid metabolism. Inhibition of AMPK leads to increased activity of acetyl-CoA carboxylase (ACC) and fatty acid synthase (FAS), enzymes involved in lipogenesis. This results in increased synthesis and accumulation of triglycerides and cholesterol in the liver, contributing to dyslipidemia. Hardie provided foundational insights into how AMPK inhibition leads to dyslipidemia in cancer patients [18]. A recent 2022 study by Hu et al. confirmed these mechanisms, showing that patients treated with cisplatin had a 30% increase in hepatic triglyceride levels due to AMPK inhibition [19]. However, the broader implications of these findings should be explored further. Specifically, there is a need to investigate whether different chemotherapeutic agents exhibit varying levels of impact on AMPK activity and whether certain patient populations are more susceptible to these effects. Longitudinal studies could provide valuable data on the persistence and reversibility of chemotherapy-induced dyslipidemia.

Chronic Inflammation

Chronic inflammation is a hallmark of chemotherapy-induced metabolic changes. Chemotherapy can activate the nuclear factor kappa B (NF-κB) pathway, a key regulator of inflammatory responses. Activation of NF-κB leads to the transcription of pro-inflammatory cytokines such as tumor necrosis factor-alpha (TNF-α), interleukin-6 (IL-6), and C-reactive protein (CRP). These cytokines contribute to systemic inflammation, which promotes insulin resistance and disrupts lipid metabolism. Balkwill and Mantovani described the role of NF-κB in chemotherapy-induced inflammation [20]. A recent 2022 study by Littman et al. demonstrated that cancer patients undergoing chemotherapy had elevated levels of TNF-α and IL-6, which were directly linked to increased insulin resistance and dyslipidemia [21]. While the activation of NF-κB is well-documented, further research should aim to understand the temporal dynamics of this activation and its long-term consequences. Moreover, studies exploring the efficacy of NF-κB inhibitors or anti-inflammatory treatments in mitigating these effects could be highly beneficial.

Oxidative Stress

Oxidative stress is another critical mechanism linking chemotherapy to metabolic syndrome. Chemotherapeutic agents, particularly anthracyclines like doxorubicin, can generate reactive oxygen species (ROS). These ROS cause oxidative damage to cellular components, including DNA, proteins, and lipids. This oxidative stress can impair mitochondrial function, leading to decreased ATP production and increased lipid accumulation. Oxidative stress also promotes inflammation and insulin resistance, both of which are critical factors in developing metabolic syndrome. De Nunzio et al. highlighted the role of oxidative stress in chemotherapy-induced metabolic syndrome [22]. A recent study found that ROS levels in patients treated with doxorubicin were 60% higher, correlating with a significant increase in markers of oxidative damage and metabolic syndrome [23]. While oxidative stress is a well-recognized consequence of chemotherapy, the specific sources and types of ROS involved need further elucidation. Additionally, investigating the role of mitochondrial dysfunction in greater detail could reveal new targets for intervention. Antioxidant therapies, both pharmacological and dietary, should be explored as potential mitigators of these adverse effects.

Fibrosis and Tissue Damage

Chemotherapy can lead to fibrosis and damage to various tissues, including the liver and adipose tissue. Fibrosis in the adipose tissue reduces its capacity to store lipids, leading to ectopic fat deposition in organs such as the liver and muscles. This ectopic fat can further exacerbate insulin resistance and dyslipidemia. Additionally, chemotherapy-induced fibrosis in vascular tissues can lead to endothelial dysfunction, increasing the risk of cardiovascular diseases associated with metabolic syndrome. Wu et al. discussed the impact of fibrosis on metabolic health in cancer patients [24]. A study showed that patients with chemotherapy-induced fibrosis had a 40% higher prevalence of metabolic syndrome, highlighting the role of tissue damage in metabolic disturbances [25]. These findings underscore the need for comprehensive studies on the mechanisms driving chemotherapy-induced fibrosis. Understanding the interplay between fibrotic processes and metabolic dysregulation could identify novel therapeutic targets. Furthermore, research into anti-fibrotic agents could offer promising avenues for preventing or reducing fibrosis-related complications in cancer patients.

Discussion

Patient Characteristics and Predictors of Metabolic Syndrome

Patient characteristics, such as older age and gender, significantly predict the development of metabolic syndrome in cancer patients. Older patients are at higher risk due to age-related metabolic changes and the cumulative burden of chemotherapy [10]. Women, especially those treated for breast cancer, show higher prevalence rates due to hormonal changes and specific chemotherapeutic agents [13]. Furthermore, pre-existing diabetes and baseline obesity exacerbate the risk, with diabetes contributing to insulin resistance and dyslipidemia and obesity being further aggravated by chemotherapy-induced weight gain [10,15].

Impact on Cancer Treatment Outcomes

The impact of metabolic syndrome on cancer treatment outcomes is profound. A study found that cancer survivors with metabolic syndrome had a twofold increased risk of developing cardiovascular diseases compared to those without metabolic syndrome [26]. Additionally, metabolic syndrome has been linked to poorer overall survival rates. A meta-analysis by Blaes et al. revealed that cancer patients with metabolic syndrome had a 50% higher mortality rate compared to those without the condition [9]. While these patient characteristics provide a framework for identifying high-risk individuals, it is crucial to delve deeper into the interplay between these factors. For instance, examining how hormonal therapies interact with chemotherapy in female patients could offer more precise risk stratification and management approaches.

Interaction of Predictors

Recent studies have highlighted how various predictors interact to compound the risk of metabolic syndrome. For example, a study found that older cancer patients with pre-existing diabetes had a threefold higher risk of developing metabolic syndrome post-chemotherapy compared to younger patients without diabetes [27]. Another study demonstrated that female breast cancer survivors who were obese before treatment had a 60% higher risk of metabolic syndrome, particularly if they also experienced significant chemotherapy-induced weight gain [21]. These interactions suggest a cumulative effect that necessitates a multifactorial approach to risk assessment and management. Developing predictive models that integrate multiple risk factors could enhance the accuracy of identifying patients at the highest risk of metabolic syndrome.

Chemotherapy Regimens and Patient Characteristics

The combination of chemotherapy regimens and patient characteristics further compounds the risk. A study showed that patients receiving anthracycline-based regimens who were also older and had pre-existing obesity were at a 70% higher risk of metabolic syndrome compared to younger, non-obese patients receiving the same treatment [28]. These findings highlight the need for personalized medicine approaches that consider the unique combination of risk factors present in each patient. Tailoring chemotherapy regimens and supportive care plans based on individual risk profiles could mitigate the development of metabolic syndrome.

Impact on Treatment Efficacy and Quality of Life

Utilizing a broader range of studies, it is evident that metabolic syndrome significantly impacts cancer treatment efficacy and patient quality of life. For instance, Belanger et al. reported that metabolic syndrome was associated with a 30% reduction in the effectiveness of chemotherapy in breast cancer patients, leading to higher recurrence rates [17]. Similarly, Doerr et al. found that the presence of metabolic syndrome exacerbated chemotherapy-related toxicities, thereby limiting the dose and duration of treatment that patients could tolerate [22]. This can result in suboptimal treatment outcomes and an increased risk of cancer recurrence. The implications of these findings are profound, suggesting that managing metabolic syndrome is critical not only for improving overall health but also for optimizing cancer treatment outcomes. Future research should explore the bidirectional relationship between metabolic syndrome and chemotherapy efficacy, potentially identifying metabolic markers that predict treatment response.

Common Chemotherapy Agents

The table below outlines common chemotherapy drugs associated with the development of metabolic syndrome. It includes the drug class, specific agents, and the primary metabolic effects each drug induces (Table 1).

**Table 1:** Common chemotherapy drugs that are associated with the development of metabolic syndrome

Drug class	Chemotherapy agents	Primary metabolic effects
Anthracyclines	Doxorubicin, daunorubicin	Insulin resistance, dyslipidemia, chronic inflammation, and oxidative stress [22]
Alkylating agents	Cyclophosphamide, ifosfamide	Insulin resistance, chronic inflammation, and lipid metabolism disruption [24]
Antimetabolites	Methotrexate, 5-fluorouracil	Insulin resistance, mitochondrial dysfunction, and chronic inflammation [10]
Platinum compounds	Cisplatin, carboplatin	Insulin resistance, dyslipidemia, and oxidative stress [19]
Taxanes	Paclitaxel, docetaxel	Insulin resistance, inflammation, and oxidative stress [6]
Vinca alkaloids	Vincristine, vinblastine	Insulin resistance and peripheral neuropathy leading to decreased physical activity [16]
Topoisomerase inhibitors	Etoposide, irinotecan	Dyslipidemia, insulin resistance, and chronic inflammation [17]

While this table provides a concise overview, it is essential to recognize the variability in patient responses to these agents (Table 1). Factors such as genetic predispositions, co-existing conditions, and concurrent medications can influence the metabolic effects of these drugs. Personalized therapeutic approaches that consider these variables may enhance treatment outcomes and reduce adverse metabolic effects.

Strategies for Managing Metabolic Syndrome

Managing metabolic syndrome in cancer patients requires a multifaceted approach that includes lifestyle interventions, pharmacological treatments, and continuous monitoring and surveillance. Understanding predictors such as chemotherapy regimens, patient characteristics, comorbid conditions, lifestyle factors, and genetic predictors is crucial for tailoring interventions. For instance, addressing unhealthy dietary habits and physical inactivity through personalized nutrition and exercise plans can mitigate the risk [29-32]. However, it is also critical to consider psychosocial factors that may affect adherence to these interventions. Providing psychological support and addressing barriers such as fatigue, depression, and lack of motivation can improve the effectiveness of lifestyle interventions.

Lifestyle Interventions

Diet and nutrition: A diet rich in fruits, vegetables, whole grains, and lean proteins, while low in saturated fats, trans fats, and refined sugars, is recommended. Such a diet helps in controlling weight, improving lipid profiles, and maintaining blood glucose levels. 

Specific dietary approaches: The Mediterranean diet, which emphasizes anti-inflammatory and antioxidant-rich foods, has been shown to reduce components of metabolic syndrome. A study found that patients adhering to the Mediterranean diet had a 20% reduction in the risk of developing metabolic syndrome [33]. Another study highlighting cancer survivors following a Mediterranean diet reported a 25% improvement in insulin sensitivity and a 15% reduction in body mass index (BMI) over a one-year period [34]. While these findings are promising, further research is needed to determine the long-term adherence and sustainability of such dietary interventions among cancer survivors. Additionally, exploring culturally appropriate dietary modifications could enhance the acceptance and effectiveness of these interventions.

Physical activity: Regular physical activity is essential for managing metabolic syndrome. Exercise improves insulin sensitivity, promotes weight loss, and enhances cardiovascular health.

American Cancer Society guidelines: The American Cancer Society recommends that cancer survivors engage in at least 150 minutes of moderate-intensity or 75 minutes of vigorous-intensity physical activity each week, along with muscle-strengthening exercises on two or more days per week [34]. A study found that breast cancer survivors who followed these guidelines experienced a 30% reduction in metabolic syndrome prevalence compared to those who did not [17]. Despite these guidelines, adherence to physical activity recommendations can be challenging for cancer survivors due to treatment-related fatigue and other physical limitations. Developing personalized exercise programs that accommodate these challenges and providing ongoing support can improve adherence and outcomes.

Pharmacological Interventions

Anti-diabetic medications: For patients with insulin resistance or hyperglycemia, anti-diabetic medications may be necessary. Metformin is often the first-line treatment for managing insulin resistance and type 2 diabetes. It works by decreasing hepatic glucose production and improving insulin sensitivity. A study by Consoli et al. reported that metformin use in cancer survivors reduced fasting glucose levels by 20% and improved HbA1c by 1.5% [35]. GLP-1 receptor agonists and SGLT-2 inhibitors can also be beneficial in managing blood glucose levels and promoting weight loss. Patients using these medications experienced a 15% reduction in body weight and a 10% improvement in glycemic control [36]. Further studies are needed to evaluate the long-term effects of these medications on cancer survivors, particularly regarding their impact on cancer prognosis and overall survival. Additionally, the cost and accessibility of these medications should be considered when developing treatment plans.

Lipid-lowering agents: To address dyslipidemia, lipid-lowering agents such as statins are commonly prescribed. Statins work by inhibiting HMG-CoA reductase, an enzyme involved in cholesterol synthesis, thus lowering low-density lipoprotein (LDL) cholesterol levels. They have also been shown to have anti-inflammatory effects, which can be beneficial in reducing the overall risk of cardiovascular diseases. A study showed that statin therapy led to a 25% reduction in LDL cholesterol and a 20% decrease in CRP levels among cancer survivors [21]. While statins are effective, potential side effects such as muscle pain and increased risk of diabetes should be monitored. Tailoring statin therapy based on individual risk profiles and tolerability can optimize benefits and minimize adverse effects.

Anti-hypertensive medications: Managing high blood pressure is critical in patients with metabolic syndrome. Several classes of anti-hypertensive medications can be used, including ACE inhibitors, angiotensin II receptor blockers (ARBs), beta-blockers, calcium channel blockers, and diuretics. The choice of medication should be based on the patient's overall health, the presence of comorbid conditions, and specific blood pressure targets. A study published in Hypertension showed that cancer survivors using a combination of ACE inhibitors and ARBs had a 35% reduction in systolic blood pressure and a 25% improvement in overall cardiovascular risk profile [37]. Considering the potential interactions between anti-hypertensive medications and chemotherapy agents, close monitoring and adjustment of treatment regimens are necessary to ensure optimal blood pressure control and minimize adverse effects.

Monitoring and Surveillance

Regular screening for metabolic syndrome components: Early detection and management of metabolic syndrome components are vital for preventing long-term complications. Regular screening for abdominal obesity, blood pressure, fasting glucose, triglycerides, and HDL cholesterol should be conducted. 

Effectiveness: These screenings should be integrated into routine follow-up care for cancer survivors to promptly identify and address any abnormalities [2]. A longitudinal study found that regular monitoring and early intervention reduced the progression of metabolic syndrome by 30% in cancer survivors [38]. Incorporating advanced screening technologies, such as continuous glucose monitoring and wearable devices for physical activity tracking, could enhance the accuracy and timeliness of monitoring.

Long-term follow-up and management: Cancer survivors require long-term follow-up care to monitor for late effects of treatment and manage chronic health conditions like metabolic syndrome. Multidisciplinary care teams, including oncologists, primary care physicians, endocrinologists, cardiologists, dietitians, and physical therapists, should collaborate to provide comprehensive care. Regular follow-up visits should include assessments of metabolic health, lifestyle counseling, and adjustments to treatment plans as needed to optimize health outcomes. A study indicated that patients receiving multidisciplinary care had a 40% improvement in metabolic health markers compared to those receiving standard care [34]. Establishing survivorship clinics that focus specifically on the long-term health of cancer survivors could improve access to comprehensive care. These clinics should be equipped to address both oncological and metabolic health needs, ensuring seamless coordination of care.

Impact of Cancer Therapy-Induced Metabolic Syndrome on Patient Outcomes

The development of metabolic syndrome as a result of cancer therapy significantly affects patient outcomes. The syndrome's various components can lead to severe complications, compromising the overall health and quality of life of cancer survivors.

Cardiovascular complications: Metabolic syndrome substantially increases the risk of cardiovascular complications in cancer patients. According to a study by Yeh et al., cancer survivors with metabolic syndrome have a twofold increased risk of developing cardiovascular diseases compared to those without metabolic syndrome [26]. Additionally, a cohort study involving 1,500 cancer survivors found that 40% of those with metabolic syndrome experienced cardiovascular events within five years post-treatment, compared to 20% of those without the syndrome [38]. Giovannucci et al. demonstrated that the presence of metabolic syndrome exacerbates the cardiotoxic effects of chemotherapy, particularly anthracyclines, leading to a 30% increase in the incidence of heart failure among affected patients [27]. This highlights the compounded risk when metabolic syndrome coexists with chemotherapy-induced cardiotoxicity. Considering these significant risks, integrating cardiology consultations and cardiac monitoring into the care plans of cancer patients with metabolic syndrome is crucial. Early interventions, such as the use of cardioprotective agents during chemotherapy, could help mitigate these risks.

Diabetes and glucose intolerance: Cancer therapy-induced metabolic syndrome significantly increases the risk of diabetes and glucose intolerance. A study found that cancer survivors with metabolic syndrome were 60% more likely to develop type 2 diabetes compared to those without the syndrome [28]. In a longitudinal study of 2,000 cancer survivors, 25% of those with metabolic syndrome developed diabetes within five years, compared to 10% of those without metabolic syndrome [38]. Meacham et al. reported that childhood cancer survivors with metabolic syndrome had a significantly higher incidence of glucose intolerance and type 2 diabetes, underlining the long-term impact of early-life cancer therapies on metabolic health [7]. Proactive diabetes management strategies, including regular screening and early pharmacological intervention, should be implemented for cancer survivors at high risk of diabetes. Additionally, lifestyle interventions tailored to individual patient needs can help prevent the onset of glucose intolerance and diabetes.

Obesity and weight management: Weight gain and obesity are common outcomes of cancer therapy-induced metabolic syndrome. A study published in Cancer (2012) found that 50% of breast cancer survivors with metabolic syndrome experienced significant weight gain, compared to 30% of those without the syndrome [29]. Additionally, a survey of 3,000 cancer survivors revealed that 35% of those with metabolic syndrome were obese, compared to 20% of those without the syndrome [36]. Kersten highlighted that obesity in cancer survivors is linked to poorer cancer prognosis and increased risk of recurrence, further emphasizing the need for effective weight management strategies in this population [29]. Addressing obesity requires a multidisciplinary approach that includes dietitians, physical therapists, and behavioral therapists. Personalized weight management programs that consider the unique challenges faced by cancer survivors, such as fatigue and treatment-related side effects, can improve adherence and outcomes.

Dyslipidemia and lipid profile alterations: Dyslipidemia, characterized by elevated triglycerides, low HDL cholesterol, and high LDL cholesterol, is a common component of metabolic syndrome induced by cancer therapy. A study by Blaes et al. found that 45% of cancer survivors with metabolic syndrome had dyslipidemia, compared to 25% of those without the syndrome [9]. In a cohort study of 1,800 cancer survivors, 50% of those with metabolic syndrome exhibited significantly altered lipid profiles, compared to 28% of those without [38]. Myte et al. discussed the impact of dyslipidemia on cardiovascular risk, showing that cancer survivors with dyslipidemia had a 25% higher risk of cardiovascular events [30]. This underscores the importance of managing lipid profiles to reduce long-term cardiovascular complications in cancer survivors. In addition to pharmacological interventions, dietary modifications and regular physical activity should be emphasized to manage dyslipidemia effectively. Exploring the role of novel lipid-lowering agents and their potential benefits for cancer survivors could also enhance treatment strategies.

Future Directions and Research Gaps

In the context of chemotherapy-induced metabolic syndrome, several research gaps and future directions are critical to improving our understanding and management of this condition.

Need for longitudinal studies: Longitudinal studies are essential to track the long-term effects of chemotherapy on metabolic health. Unlike cross-sectional studies, longitudinal research can provide insights into how metabolic syndrome develops and progresses over time in cancer survivors. For example, the ongoing study by Pluimakers et al. is tracking metabolic health in breast cancer survivors over 10 years, aiming to identify critical periods for intervention and how different chemotherapy regimens impact long-term metabolic outcomes [31]. Additionally, the Childhood Cancer Survivor Study (CCSS) is a large-scale longitudinal study that has been instrumental in understanding the late effects of cancer treatment, including the development of metabolic syndrome in childhood cancer survivors [32]. These studies should be expanded to include diverse populations to understand the variability in metabolic outcomes across different ethnic and socioeconomic groups. Moreover, integrating biomarker analyses in longitudinal studies could help identify early indicators of metabolic syndrome.

Mechanistic research: There is a need for more detailed mechanistic research to uncover the specific molecular and cellular pathways through which chemotherapy induces metabolic syndrome. Recent advances in this area include a study by Belanger et al. that explores the role of mitochondrial dysfunction and oxidative stress in chemotherapy-induced insulin resistance [17]. Understanding how chemotherapy disrupts insulin signaling, lipid metabolism, and inflammatory processes at a molecular level can lead to the discovery of new therapeutic targets. This research should also consider genetic and epigenetic factors that may influence individual susceptibility to metabolic changes post-chemotherapy. Developing animal models that accurately replicate human metabolic responses to chemotherapy could provide deeper insights into these mechanisms. Additionally, leveraging advanced technologies such as CRISPR and single-cell sequencing could unravel the complex interactions at the cellular level.

Personalized medicine approaches: Personalized medicine aims to tailor interventions based on individual risk factors and treatment responses. Identifying biomarkers that predict the risk of developing metabolic syndrome can enable personalized treatment plans, minimizing adverse metabolic outcomes. A study by Hu et al. used machine learning models to predict metabolic syndrome risk based on genetic, clinical, and lifestyle factors in cancer survivors, demonstrating the potential of personalized approaches [19]. Integrating predictive modeling and machine learning can further refine these personalized approaches, ensuring that interventions are specifically designed to mitigate risks for each patient. Collaboration between oncologists, endocrinologists, and data scientists is essential to develop and validate these predictive models. Incorporating real-world data from electronic health records could enhance the accuracy and applicability of these models in clinical practice.

Integrative therapies and holistic management: Managing chemotherapy-induced metabolic syndrome requires a holistic approach, including integrative therapies such as diet, exercise, and behavioral interventions. Complementary therapies like acupuncture, yoga, and mindfulness have been shown to improve metabolic health and overall quality of life. For instance, a randomized controlled trial by Littman et al. found that a combined intervention of yoga and dietary counseling significantly reduced components of metabolic syndrome in breast cancer survivors [21]. Developing multidisciplinary care models that incorporate oncologists, endocrinologists, dietitians, physical therapists, and mental health professionals is crucial for providing comprehensive care to cancer survivors. Future research should focus on the long-term efficacy and sustainability of these integrative therapies. Exploring the synergistic effects of combining multiple complementary approaches could provide more effective strategies for managing metabolic syndrome. Additionally, patient education and engagement are critical for the success of holistic management programs.

## Conclusions

The review highlights the need for a comprehensive approach to managing metabolic syndrome in cancer survivors. Personalized care plans should take into account factors such as chemotherapy regimens, patient characteristics, and lifestyle choices. Effective management includes lifestyle interventions, medications, and regular monitoring. Research should continue to explore the molecular pathways involved and track metabolic health over time. Integrating dietary counseling, physical activity, and complementary therapies can improve metabolic health and quality of life. A multidisciplinary approach can help healthcare providers address the unique risks faced by cancer survivors and enhance long-term health outcomes.
